# Genetic Diversity and Population Structure Analysis of *Triticum aestivum* L. Landrace Panel from Afghanistan

**DOI:** 10.3390/genes12030340

**Published:** 2021-02-25

**Authors:** Muhammad Massub Tehseen, Deniz Istipliler, Zakaria Kehel, Carolina P. Sansaloni, Marta da Silva Lopes, Ezgi Kurtulus, Sana Muazzam, Kumarse Nazari

**Affiliations:** 1 Department of Field Crops, Ege University, Bornova, Izmir 35100, Turkey; massub.tehseen@gmail.com (M.M.T.); deniz.istipliler@ege.edu.tr (D.I.); 2 International Center for Agricultural Research in the Dry Areas (ICARDA), ICARDA-PreBreeding & Genebank Operations, Rabat 10000, Morocco; Z.Kehel@cgiar.org; 3 International Maize and Wheat Improvement Center (CIMMYT), Carretera México-Veracruz Km. 45, El Batán, Texcoco C.P. 56237, Mexico; C.Sansaloni@cgiar.org; 4 IRTA (Institute for Food and Agricultural Research and Technology), 25198 Lleida, Spain; marta.dasilva@irta.cat; 5 International Center for Agricultural Research in the Dry Areas (ICARDA), Turkey-ICARDA Regional Cereal Rust Research Center (RCRRC), Menemen, Izmir 35661, Turkey; E.Kurtulus@cgiar.org; 6 Department of Plant Breeding and Genetics, University of Agriculture Faisalabad, Faisalabad 38000, Pakistan; sanamuazzam@ymail.com

**Keywords:** Afghan wheat landraces, genetic diversity, population structure

## Abstract

Landraces are a potential source of genetic diversity and provide useful genetic resources to cope with the current and future challenges in crop breeding. Afghanistan is located close to the centre of origin of hexaploid wheat. Therefore, understanding the population structure and genetic diversity of Afghan wheat landraces is of enormous importance in breeding programmes for the development of high-yielding cultivars as well as broadening the genetic base of bread wheat. Here, a panel of 363 bread wheat landraces collected from seven north and north-eastern provinces of Afghanistan were evaluated for population structure and genetic diversity using single nucleotide polymorphic markers (SNPs). The genotyping-by-sequencing of studied landraces after quality control provided 4897 high-quality SNPs distributed across the genomes A (33.75%), B (38.73%), and D (27.50%). The population structure analysis was carried out by two methods using model-based STRUCTURE analysis and cluster-based discriminant analysis of principal components (DAPC). The analysis of molecular variance showed a higher proportion of variation within the sub-populations compared with the variation observed as a whole between sub-populations. STRUCTURE and DAPC analysis grouped the majority of the landraces from Badakhshan and Takhar together in one cluster and the landraces from Baghlan and Kunduz in a second cluster, which is in accordance with the micro-climatic conditions prevalent within the north-eastern agro-ecological zone. Genetic distance analysis was also studied to identify differences among the Afghan regions; the strongest correlation was observed for the Badakhshan and Takhar (0.003), whereas Samangan and Konarha (0.399) showed the highest genetic distance. The population structure and genetic diversity analysis highlighted the complex genetic variation present in the landraces which were highly correlated to the geographic origin and micro-climatic conditions within the agro-climatic zones of the landraces. The higher proportions of admixture could be attributed to historical unsupervised exchanges of seeds between the farmers of the central and north-eastern provinces of Afghanistan. The results of this study will provide useful information for genetic improvement in wheat and is essential for association mapping and genomic prediction studies to identify novel sources for resistance to abiotic and biotic stresses.

## 1. Introduction

Wheat (*Triticum aestivum* L.) is the primary source of calorie intake for more than one third of the global population and is ranked as the third most important crop in the world [1,2]. The common hexaploid bread wheat was domesticated around 8000 years ago in the Fertile Crescent [3]. There has been an overall increase in wheat production worldwide over the years; however, countries like Afghanistan have seen steady decline in wheat production, which is alarming in regions already struggling economically and politically for stability and to meet food demand [2]. Today, wheat plays a critical role in the fight against food security; however, in order to achieve high production goals, most of the breeding programmes have started utilizing naturally existing diversity deemed fit for a specific agro-climatic region or are simply relying on germplasm developed by private and international research organizations. This has led to narrowing down the genetic diversity from a broader gene pool. Such narrow genetic diversity poses a serious threat to sustainable production, which is crucial for the fast-growing global population and dramatically changing climatic conditions [4]. Wheat landraces are considered the only primary gene pool for bread wheat and for hexaploid bread wheat. The importance is more significant because of its hybrid origin and it does not share a direct link to an ancestor with similar genetic constituents [5]. Therefore, to widen the already available gene pool, landraces have been used in wheat breeding programmes and have achieved success in introducing disease resistance, quality, and other useful agronomic traits into elite lines and cultivars [5,6,7].

Landraces are defined as locally adapted distinct species with higher levels of tolerance to both abiotic and biotic stresses, resulting in moderate to high yield with low to zero input agricultural conditions [8,9]. Landraces are considered to have evolved as a combination of both natural and artificial selection over time and they adapt not only to the crops’ centres of origin but also to new geographic regions following introduction and transplantation. Landraces play an important role in modern plant breeding by transferring various key traits to cultivars and have been used in breeding programmes of several crops including maize, legumes, rice, and wheat [10,11,12,13,14,15].

Afghanistan lies close to the centre of origin of hexaploid wheat and enjoys rich topography, rich history, and a complex tribal system. It is considered the third largest centre of origin for many domesticated crops worldwide and has played crucial role in the domestication of many crops such as barley (*Hordeum vulgare*), peas (*Pisum sativum*), rye (*Secale cereal*), and chickpeas (*Cicer arietinum*) [16]. During the past years, several factors, including frequent armed conflicts, have resulted in the loss of all of the known germplasm collection [2]; in addition, only a few studies with small to moderate populations have investigated the population structure and genetic diversity of the Afghan wheat landraces [2,5,17,18,19,20]. The Afghan bread wheat population used in the present study is conserved and maintained in the gene bank of the International Centre of Agricultural Research in Dry Areas (ICARDA) and is composed of 363 accessions collected from seven north and north-eastern provinces of Afghanistan. These Afghan wheat accessions are considered as landraces based on their collection time, location, and information. However, so far, no genetic assay has been applied before to confirm the genetic basis of these wheat accessions.

The recent advances in the development of molecular markers and high-throughput systems have generated a wealth of information for a large number of plants [21,22,23]. Single nucleotide polymorphisms (SNPs) are the most common variations in the genome sequence [2,24], making them well suited for studies like genome wide association studies (GWAS) [25], genomic selection [26], population structure, and genetic diversity [22], requiring a large number of markers. Thus, application of SNPs markers enables rapid genotyping with low error rates, as they occur on the genome with high frequency [24]. The objective of the present study was to examine a large collection of Afghan bread wheat landraces to unravel the population structure and genetic diversity among the landraces and the sub-populations. Understanding the genetic diversity and population structure of these landraces will aid in the utilization of these bread wheat landraces in future breeding programmes in order to enhance the genetic base of hexaploid wheat and identify new genomic regions/sources of resistance and economically useful traits, and finally to deploy those regions in elite wheat breeding programmes.

## 2. Results

### 2.1. SNP Marker Distribution

**Figure 1:**
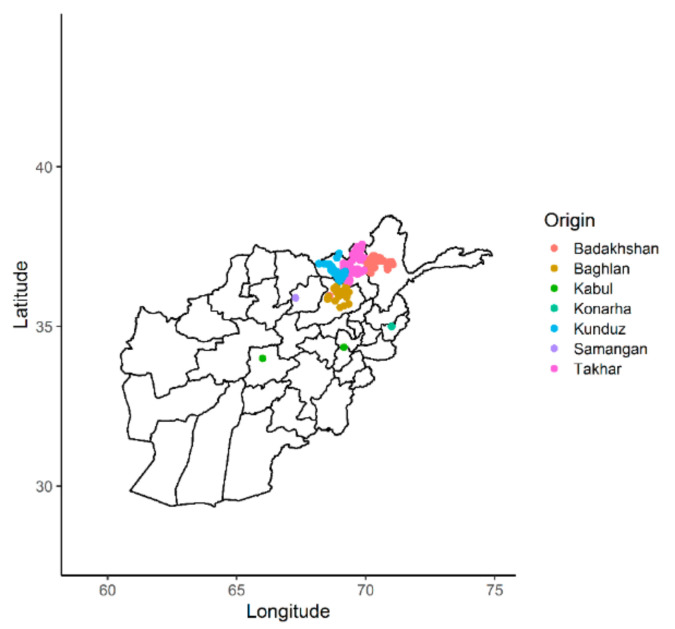
Distribution of the Afghan landraces according to their geographic origins.

**Figure 2:**
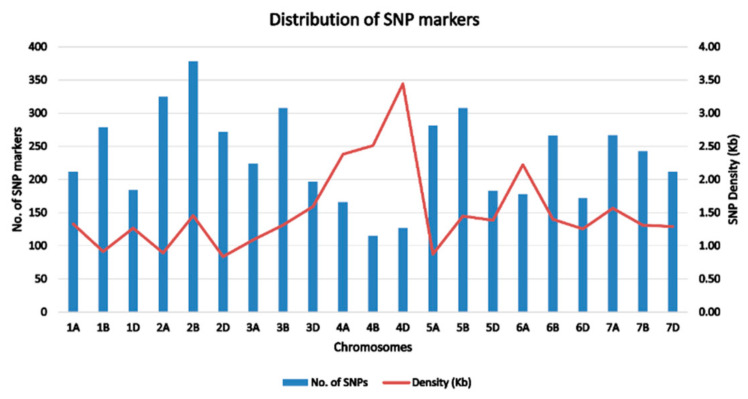
Distribution and the corresponding single nucleotide polymorphism (SNP) densities of 4897 SNPs across 21 chromosomes of bread wheat landraces from Afghanistan.

A total of 363 bread wheat landraces collected from seven provinces of Afghanistan were genotyped using Diversity Arrays technology (DArT). One of the landraces from Kabul did not correspond to the recorded geographic location and therefore has been either recorded incorrectly or is a mixture (Figure 1). A set of 23,079 SNPs was generated and, after quality control and SNP filtering, a set of 4897 high-quality SNPs with known chromosomal positions on the 21 chromosomes of wheat were used in the final analysis. The highest number of markers were mapped on the B genome (1897), followed by the A genome (1653) and the D genome (1347). The average density of markers across the whole genome was 1.51 markers per kb (Table 1; Figure 2). The highest number of markers was found on chromosome 2B (378), followed by chromosome 2A (325) and chromosomes 3B and 5B (308). The lowest number of markers was found on chromosome 4B (115). The lowest marker density was observed on chromosome 4D (3.44 kb) among all the chromosomes, whereas the highest marker density was observed on chromosome 2D (0.84 kb). Average marker densities on the A, B, and D genomes were 1.54 kb, 1.53 kb, and 1.55 kb, respectively.

### 2.2. Population Stratification and Genetic Relationships

**Figure 3:**
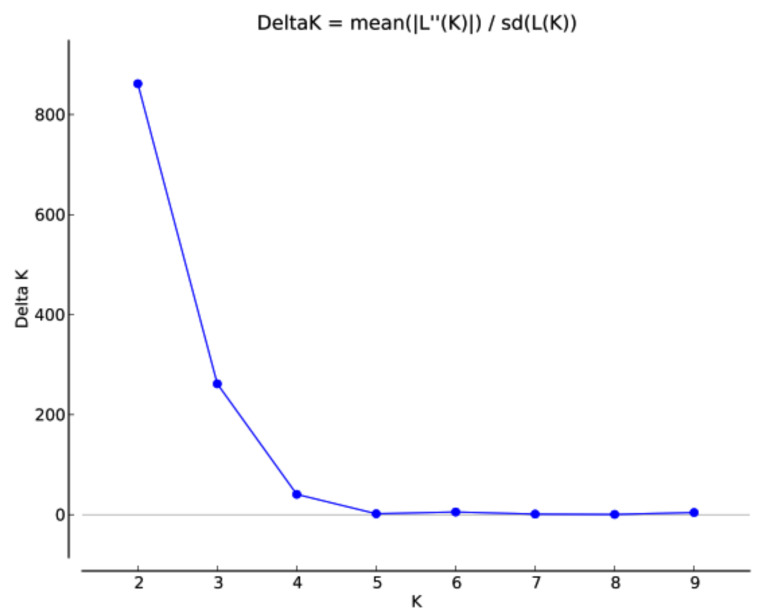
Population structure of the 363 Afghan bread wheat landraces.

The optimal population stratification was measured using two approaches. The first approach was model-based as implemented in the STRUCTURE program. The optimum number of sub-populations was inferred based on the rate of change of likelihood (Δ*K*). The results indicated that the population structure was optimum at *K* = 2. In the plot of *K* against Δ*K*, there was a steep slope at *K* = 2 following the flattening of the curve (Figure 3.) Population structure showed higher proportions of admixture. However, the landraces with close geographic proximities like Badakhshan and Takhar were clustered together, whereas Baghlan and Kunduz showed more similarity and were grouped together. In the two sub-populations identified by the STRUCTURE algorithm, there was no clear differentiation amongst the landraces. The accessions from Kabul, Konarha, and Samangan were clustered in Sub-population 2, whereas the landraces from Badakhshan, Baghlan, Kunduz, and Takhar were distributed in both sub-populations (Table S2). Among the two sub-populations, 238 landraces were grouped in Sub-population 1, while 125 landraces were grouped in Sub-population 2. The fixation index (Fst) estimated by the STRUCTURE results suggested significant divergence within the two sub-populations (Table 2). An Fst value of 0.51 and 0.14 was obtained for Sub-population 1 and Sub-population 2, respectively.

**Figure 4:**
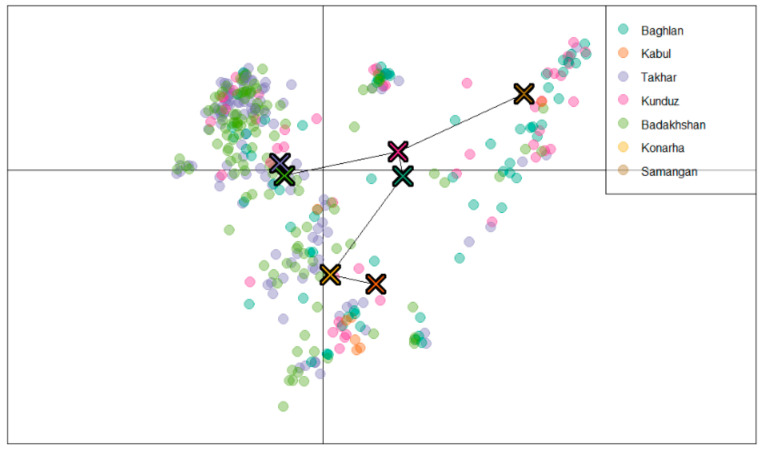
Inference of the sub-populations by discriminant analysis of the principal components (DAPC) analysis grouping landraces from different regions together.

The second approach was based on discriminant analysis of the principal components (DAPC) and the results showed at least three sub-populations (Figure 4). Hence, the landraces were grouped into three sub-populations with 175, 117, and 71 landraces in Sub-populations 1, 2, and 3 respectively. Sub-population 1 comprised landraces from Baghlan, Takhar, Kunduz, and Badakhshan. Landraces from Kabul, Konarha, and Samangan were grouped together in Sub-population 2, and Sub-population 3 contained 71 landraces; the majority of them were from Takhar (25) and Badakhshan (23). The DAPC analysis clustered the landraces from Badakhshan and Takhar together, those from Baghlan and Kunduz together, and those from Kabul and Konarha together (Figure 4).

**Figure 5:**
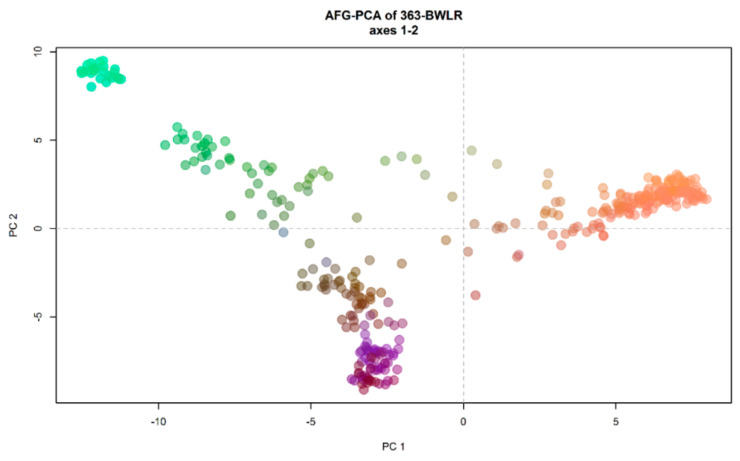
Principal component analysis (PCA) of 363 Afghan bread wheat landraces. colours are coded as the first three principal components (PCs) of the PCA recorded, respectively, on the red, green, and blue channel.

The results of principal component analysis (PCA) is in accordance with the DAPC analysis and showed three clusters. The first, second, and third PCs explained 18.76%, 10.69%, and 4.34% of the total variation, respectively (Figure 5).

**Figure 6:**
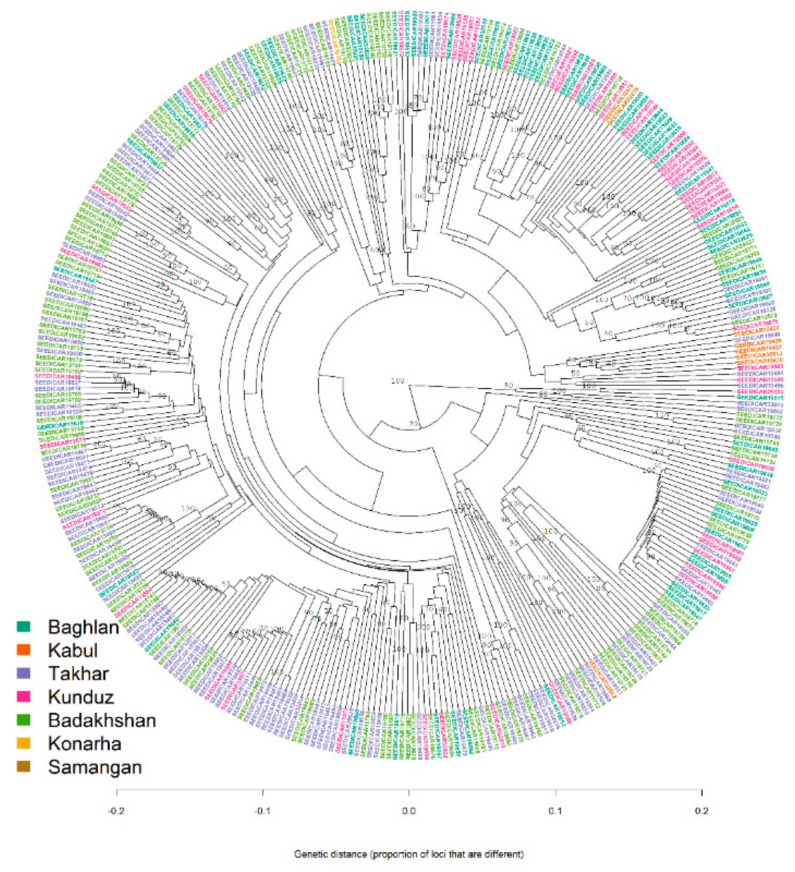
Neighbour-joining clustering of 363 Afghan bread wheat landraces.

**Figure 7:**
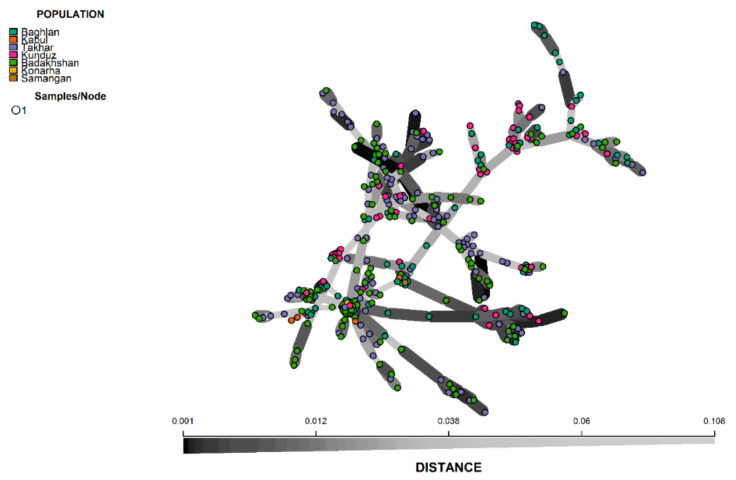
Minimum spanning network (MSN) of 363 Afghan bread wheat landraces.

It is to be noted that both the STRUCTURE and DAPC analysis clustered the population with high levels of admixture, which was evident from the neighbour-joining based clustering and minimum spanning network (MSN) (Figure 6 and Figure 7). The neighbour-joining-based clustering and MSN showed the landraces clustered with high admixture and the clustering of the landraces tend to be based on their geographic origin, as the landraces from Badakhshan, Takhar, Baghlan, and Kunduz were mostly grouped together.

### 2.3. Genetic Differentiation among Sub-Populations

The analysis of molecular variance (AMOVA) based on the STRUCTURE results revealed that there was very small genetic variation (0.29%) between the two sub-populations. However, greater genetic variance was found within the two sub-populations (99.71%) (Table 2). The same results were identified with clustering based on DAPC and geographic origin, with genetic variance of 2.4% and 4.4%, respectively, between the sub-populations derived from DAPC and by geographic background. These results suggest that the landraces from Afghanistan shared a common ancestry and are highly admixed, with variation within the sub-populations from STRUCTURE, DAPC, and origin-based analysis were 99.71%, 97.53%, and 95.58%, respectively (Table 3). In order to explore the levels of diversity among the sub-populations, genetic distances among the sub-populations were also calculated (Table 4). The difference among the two sub-populations clustered by STRUCTURE reflected a genetic distance of 0.004, whereas a maximum genetic distance of 0.023 in the sub-populations from DAPC analysis was found between sub-population 1 and sub-population 2. The clustering with respect to the geographic origins of the landraces showed the maximum genetic distance of 0.399 between the landraces from Konarha and Samangan, followed by a genetic distance of 0.308 between landraces from Kabul and Konarha. The minimum genetic distance of 0.003 was estimated between the landraces from Badakhshan and Takhar.

### 2.4. Genetic Diversity across Sub-Populations

The mean values of different alleles (*Na*) and the number of effective alleles (*Ne*) of the two sub-populations identified by STRUCTURE were 1.999 and 1.591, respectively (Table 5), and the mean values for overall population for Shannon’s Index (*I*), the diversity index (*He*), and the unbiased diversity index (*uHe*) were 0.516, 0.345, and 0.346, respectively. Between the two sub-populations grouped by STRUCTURE, Sub-population 1 (*I* = 0.518, *He* = 0.346, and *uHe* = 0.348) showed higher diversity than Sub-population 2 (*I* = 0.514, *He* = 0.343, and *uHe* = 0.345). Sub-population 2 clustered by the DAPC approach showed higher diversity (*I* = 0.517, *He* = 0.345, and *uHe* = 0.347) compared with Sub-population 1 and Sub-population 3. The overall mean values of the genetic indices for the populations derived from the DAPC approach were *Na* = 1.994, *Ne* = 1.579, *I* = 0.506, *He* = 0.338, and *uHe* = 0.341. When the landraces were grouped based on their geographic origin, the landraces from Badakhshan province showed the highest genetic diversity (*I* = 0.509, *He* = 0.340, and *uHe* = 0.343), whereas the landraces from Kabul showed the least genetic diversity (*I* = 0.304, *He* = 0.0236, and *uHe* = 0.231). Since there was only one landrace accession from Konarha and Samangan, their genetic indices were not calculated and hence a comparison with other provinces was not possible.

### 2.5. Clustering via Geographic Origin

**Figure 8:**
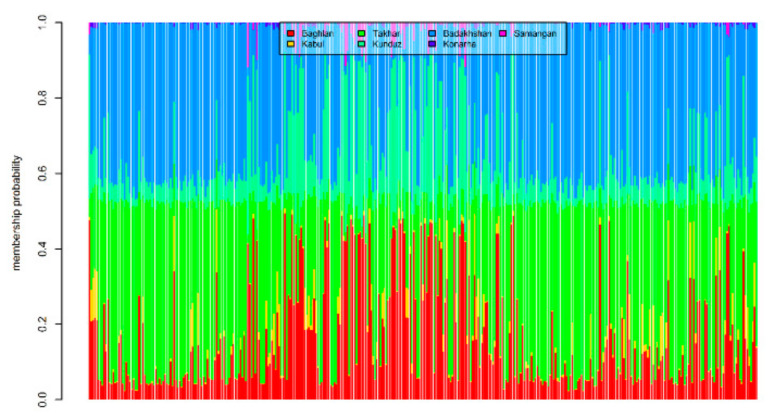
Estimated population membership probability of 363 bread wheat landraces from seven states of Afghanistan, where each bar represents a landrace.

The two methods used in the study to assess the population structure clustered the landraces with close proximity to each other together, but with a high proportion of admixture. For example, in the DAPC method, 72 landraces from Badakshan province were grouped in Sub-population 1, while 24 and 23 landraces of the same origin were grouped in Sub-population 2 and Sub-population 3, respectively. Similarly, 40 landraces from Baghlan province were clustered in Sub-population 2, while the rest divided into Sub-population 1 (12) and Sub-population (3). The majority of the landraces from Takhar province were grouped into Sub-population 1 (77), the rest were grouped into Sub-population 3 (25) and Sub-population 2 (17). Six out of seven landraces collected from Kabul province were grouped into Sub-population 2, along with the two landraces from Konarha and Samangan province, whereas one landrace from Kabul province was grouped into Sub-population 3. A similar trend was observed when the whole panel was divided into two sub-populations using the STRUCTURE method, where the landraces from Badakhshan, Takhar, Kunduz, and Baghlan were assigned to both the sub-populations. However, the landraces from Kabul, Konarha, and Samangan were all grouped in Sub-population 2. In the overall trend, it was observed that the majority of the landraces from the neighbouring states were slightly favoured to group together than with the landraces from the provinces which are far away (Figure S1). However, based on the bar plot of the membership coefficient of the 363 landraces obtained from DAPC analysis, it was observed that the landraces from the north and north-eastern states of Afghanistan are highly admixed (Figure 8).

## 3. Discussion

The potential use of wheat landraces for both abiotic and biotic stress resistance has been given significant importance in many breeding programmes [27,28,29,30,31]. Genetic diversity analysis is a stepping stone towards the development of high-yielding germplasm in order to meet the food security requirements of the world’s increasing population [32]. The current study was planned to provide valuable information on the population structure and genetic diversity of 363 bread wheat landraces collected from seven provinces of Afghanistan, which could potentially be used to broaden the genetic base of Afghan wheat germplasm. It will also subsequently help in genome-wide association study (GWAS) in order to identify novel genomic regions associated with resistance against abiotic and biotic stresses.

In this study, a set of approximately 5000 genotyping-by-sequencing (GBS) derived high-quality SNPs were used, obtained from 363 bread wheat landraces. This research shows the usefulness of GBS-derived markers for population structure and genetic diversity studies. In the current study, the B genome had the highest number of SNPs, followed by the A and D genomes, which is in agreement with many previous studies [33,34]. Generally, in previous studies of wheat genetic diversity and population studies, the number of markers on the A and/or B genomes was many times higher than in the D genome [33,34,35,36]. However, in the present study, the number of markers on the D genome was slightly less than those on the A and B genome and, more interestingly, the D genome had the highest marker density per kb. This indicates that these landraces possess higher sequence diversity in the D genome compared with other sources. Greater sequence diversity in the D genome may indicate the presence of novel sources of genes that could be effectively utilized in elite wheat breeding programmes around the world [37]. Reduction in the D genome bottleneck will broaden the genetic base and enhance the rate of genetic gain and, as a result, will help in protecting wheat from ever-changing adverse climatic conditions and current limited genetic variation for the useful economical traits [4].

### 3.1. Population Structure

Population stratification analysis is useful for understanding genetic diversity and also facilitates GWAS analysis [38]. In the mapping studies, the presence of genetic structure within population can lead to false positives; therefore, special emphasis is given to carefully analysing the underlying population structure of any population to be used for marker–trait associations [39]. Therefore, analysis of population structure is considered the first step to conduct GWAS for true marker–trait associations [32,40]. In our study, the STRUCTURE results suggested the population could be clustered in two sub-populations. The clustering was mildly in accordance with the geographic background of the landraces. The two clusters were highly admixed and suggested that most of the variation is among the landraces within the clusters. Although two sub-populations may be reasonable for our panel, based on the population size and the variation in the number of landraces representing the seven provinces from which they were collected, we were still a bit sceptical about the results from STRUCTURE. It has been reported that the optimal *k* = 2 value sometimes misrepresents the true structure in the population and could possibly mean that either there is no definite population structure, or the STRUCTURE program failed to successfully identify the underlying genetic structure of the collection [41]. In addition to STRUCTURE, we used DAPC, PCA, neighbour-joining cluster analysis, and MSN analysis to truly understand the genetic structure of this population. Based on the results of these analyses, it was observed that there are at least three sub-populations in the population. The results of DAPC and PCA also contained admixture within the clusters, yet this meets our expectations in a better way, as it was observed that the landraces from the neighbouring provinces were mostly clustered together as compared with the two sub-populations derived by STRUCTURE. Based on the DAPC results, the landraces from Badakhshan and Takhar were grouped together, the landraces from Baghlan and Kunduz were grouped together, and the landraces from Kabul and Konarha were genetically closer to each other. The provinces of Badakhshan, Takhar, Baghlan, and Kunduz belong to one agro-ecological zone of Afghanistan (i.e., north-eastern), with similar overall climate conditions except for annual average precipitation, which is significantly higher in Badakhshan and Takhar compared with Baghlan and Kunduz, which may contribute to the genetic similarity observed in the study between the landraces from Badakhshan and Takhar and between the landraces from Baghlan and Kunduz (Table S3). Furthermore, the high levels of overall admixture between the landraces as revealed by all the methods used in the study may also be attributed to the free exchange of seeds between farmers of the north-eastern states of Afghanistan. This is in agreement with previous studies reporting high admixture and low genetic variation among the Afghan landraces [5,42,43]. A previous study of the population structure and genetic diversity of diverse 446 Afghan landraces collected from 17 provinces and different agro-climatic regions showed higher genetic diversity compared with our study [2]. However, this can be attributed to the diverse collection from distant provinces, as the genetic structure of the landraces from Badakshan, Takhar, and Baghlan was similar to our study, indicating that the higher diversity of Afghan landraces in the previous study is due to the fact that the landraces were collected from provinces far from each other, whereas in the present study, the landraces were primarily collected from adjacent provinces.

### 3.2. Genetic Differentiation of Populations

Genetic differentiation due to population structure is measured by Fst. An Fst value ranges from 0 to 1 and a value of 0.15 is considered significant in distinguishing the populations [44]. Thus, significant differentiation was observed in both sub-populations found by STRUCTURE. This is in accordance with the AMOVA results, where most of the variation (99.71%) was accounted for within sub-populations. Similarly, when the population was clustered into three sub-populations, as was indicated by DAPC analysis, the majority of the variation (97.53%) was observed within the population and only a small fraction (2.47%) of the variation was accounted for between the three sub-populations. When the geographic origin was used as a proxy for clustering the landraces, a similar trend was observed and 95.58% of the total variation was observed within populations. Our results are in agreement with the previous study, which also observed higher genetic variation within the landraces of the sub-populations [2]. The DAPC analysis clustered the population into three sub-populations, in which majority of the landraces from Badakhshan and Takhar provinces clustered together in Sub-population 1 and the majority of the landraces from Baghlan and Kunduz provinces were grouped into Sub-population 2. These results closely resemble existing micro-climatic conditions within the north-eastern agro-climatic zone of Afghanistan. Genetic distance analysis was used to identify the difference among the provinces; the strongest correlation was observed between landraces from Badakhshan and Takhar, again in agreement with the results observed by DAPC analysis. The highest genetic distances were observed in landraces from Konarha and Samangan (0.399), and Kabul and Konarha (0.308); however, even these distances are not significant, which implies that due to the narrow agro-climatic zone of the landraces collected from the seven provinces of Afghanistan, there are high proportions of admixture among them and the differences between the landraces is due to their geographic background. The same findings were observed in the previous studies, where landraces from Badakshan and Takhar showed higher levels of similarity and the landraces were differentiated on the basis of geographic origin and agro-climatic zones [2,5,30].

### 3.3. Genetic Diversity Indices

The allelic patterns and indices of genetic diversity shed light on the genetic diversity within sub-populations. Clustering based on STRUCTURE observed slightly higher genetic diversity in Sub-population 1 compared with Sub-population 2, which could be attributed to the size of Sub-population 1. In the case of three sub-populations, as grouped by DAPC analysis, Sub-population 2 showed the highest genetic diversity. Sub-population 2 comprised mainly landraces from Baghlan and Kunduz, identifying the highest genetic diversity amongst the landraces from these regions. The landraces from Baghlan also showed significant genetic diversity in an earlier study, thus confirming our results [5]. Interestingly, when the landraces were clustered based on their geographic background, the highest genetic diversity was observed in landraces from Badakhshan province. This may be attributed to the high number of accessions from Badakhshan. However, similar findings were reported in a former study, which indicated the highest genetic diversity among the landraces from Badakhshan province when compared with landraces from nine other provinces in Afghanistan [2]. Badakhshan was followed by Takhar and Baghlan provinces. Although the results suggested that, however, there is low genetic variation between the sub-populations of different provinces in north and north-eastern provinces of Afghanistan, there exists considerably high genetic diversity in landraces within the provinces and sub-populations. The understanding of diversity within the sub-populations of landrace populations from Afghanistan will improve future planning collecting missions to gather more diversity, optimize conservation strategies for landraces from Afghanistan, and efficiently provide valuable traits and alleles needed in wheat breeding programmes.

## 4. Materials and Methods

### 4.1. Plant Material

Three hundred and sixty-three Afghan bread wheat landrace accessions assembled from 7 north and north-eastern states of Afghanistan were used in the study. All landraces are conserved in the International Center for Agricultural Research in the Dry Areas (ICARDA) gene bank. The highest numbers of landraces were collected from Badakhshan and Takhar regions, with 119 accessions from each region. The rest of the accessions were collected from Baghlan (*n* = 66), Kabul (*n* = 7), Konarha (*n* = 1), Kunduz (*n* = 50), and Samangan (*n* = 1). Badakhshan, Baghlan, Takhar, and Kunduz belong to one agro-ecological zone (i.e., north-eastern), whereas Kabul, Samangan, and Konarha belong to three different agro-ecological zones, which are central, north and east, respectively. Details of the landraces used in the study are presented in the Table S1.

### 4.2. Genotyping

The landraces were genotyped by the high-throughput genotyping by sequencing (GBS) method using DArT technology [45] at the Genetic Analysis Service for Agriculture (SAGA) of the International Maize and Wheat Improvement Center (CIMMYT) in Mexico, supported by the Seed of Discovery Project, SeeD. Markers were filtered based on missing data >20%, minor allele frequency (MAF) < 5%, and other parameters such as call rate, polymorphic information content (PIC), and reproducibility. In the final set, only the markers with known chromosomal location and position were retained, which resulted in 4897 high-quality SNP markers to be used in the study.

### 4.3. Population Structure Analysis

In order to analyse the underlying population structure in the Afghan landraces, STRUCTURE software (v.2.3.4) was used, which implements model-based Bayesian cluster analysis [46]. The program was run for 10 independent replicates for each putative sub-population ranging from *k* = 2–10 under the admixture model of population structure and was assessed with a burn-in period of 50,000 and 50,000 Markov Chain Monte Carlo (MCMC) replications. To infer the optimal clusters/sub-populations, the best *K* value representing the optimum number of sub-populations was estimated as Delta *K* (Δ*K*) based on the change in the log probability of data between successive structure iterations using Structure Harvester [47]. For the optimal *K* value, membership coefficient matrices of three replicates from the STRUCTURE analysis were used in CLUMPP [48] to generate both individual and population Q-matrices, which after integration of the geographical information, were used to create bar plot using DISTRUCT program [48].

The second approach to analyse the population structure was based on the discriminant analysis of principal components (DAPC), which was implemented using an R package, “adegenet” [49]. DAPC uses *K*-means clustering of principal components to identify groups of individuals. It is run with different numbers of clusters, each of which gives rise to a statistical model and an associated likelihood. The best supported model, and therefore the number and nature of clusters, it is assessed using the Bayesian Information Criterion (BIC).

### 4.4. Genetic Diversity and Analysis of Molecular Variance

In order to analyse the genetic variation amongst the 363 bread wheat landraces from Afghanistan, various diversity parameters like the number of different alleles (*Na*), the number of effective alleles (*Ne*), Shannon’s index (*I*), the diversity index (*He*), the unbiased diversity index (*uHe*), and the percentage of polymorphic loci (PPL) were measured using GenAlex v6.503 [50]. The number of sub-populations defined by STRUCTURE and DAPC, and sub-populations based on the geographic origin of the landraces were used for AMOVA and the calculations of Nei’s genetic identity and genetic distance among populations using GenAlex v6.503 [50]. For the estimation of genetic relationship among landraces, Principal Component Analysis (PCA) was estimated using the R package “adegenet” [51] in R studio [52]. A neighbour-joining phylogenetic tree based on a simple matching dissimilarity coefficient without the assumption of an evolutionary hierarchy and a minimum spanning network (MSN) was constructed using the r package “poppr” [53].

## 5. Conclusions

This study provided a detailed view of the population structure and genetic diversity of 363 bread wheat landraces from seven provinces of Afghanistan. In this study, SNPs obtained from high-throughput GBS technology were used to explore population structure and genetic diversity of bread wheat landraces and suggests that these markers with high genome coverage are useful for revealing the genetic diversity and population structure. The population structure analysis revealed that the landraces originating from different geographic regions of north and north-eastern parts of Afghanistan were best divided into three distinct groups based mainly on geographical background and micro-climatic conditions within the agro-ecological zones of the landraces. However, high proportions of admixture were also observed, revealing the possibility of historical free seed exchange among farmers within these provinces. Further, it was observed that the genetic diversity was higher within the landraces compared with between the sub-populations, which is unique to these particular landraces and has been previously reported as well. The study also found out that the number of markers on the D genome, interestingly, was nearly similar to those on the A and B genomes, which is also unique to these landraces, as previous studies on bread wheat reported that the D genome had 2–5 times fewer markers than the A and B genomes. This indicated high sequence diversity present in the D genome in the Afghan landraces. Thus, this suggests that this diversity can be utilized in the elite wheat breeding programmes to widen the genetic base of the overall breeding populations. This knowledge of the genetic diversity and population structure of landraces from central and north-eastern provinces of Afghanistan will be important for future studies in the region, conferring resistance to abiotic and biotic stresses from GWAS to identify and isolate marker–trait associations for important economical traits.

## Figures and Tables

**Table 1:** Genomic distribution of 4897 SNPs mapped on 21 hexaploid wheat chromosomes.

Chromosome	No. of SNPs	SNP Percentage	Start Position	End Position	Length (kb)	Density (kb)
1A	212	4.33	1181	281,698	280.5	1.33
1B	279	5.7	604	254,989	254.4	0.91
1D	184	3.76	2442	233,037	230.6	1.27
2A	325	6.64	833	290,168	289.3	0.89
2B	378	7.72	593	550,308	549.7	1.46
2D	272	5.55	262	227,366	227.1	0.84
3A	224	4.57	105	243,683	243.6	1.09
3B	308	6.29	179	404,300	404.1	1.31
3D	197	4.02	798	313,780	313.0	1.59
4A	166	3.39	282	395,099	394.8	2.38
4B	115	2.35	1793	288,834	287.0	2.51
4D	127	2.59	2043	437,139	435.1	3.44
5A	281	5.74	689	245,844	245.2	0.87
5B	308	6.29	173	445,996	445.8	1.45
5D	183	3.74	1657	253,399	251.7	1.38
6A	178	3.63	53	395,985	395.9	2.22
6B	266	5.43	404	372,421	372.0	1.40
6D	172	3.51	1168	215,761	214.6	1.25
7A	267	5.45	333	418,101	417.8	1.57
7B	243	4.96	2096	318,562	316.5	1.31
7D	212	4.33	888	273,554	272.7	1.29

**Table 2:** The STRUCTURE results of 363 Afghan landraces for the fixation index (Fst), average distances (expected heterozygosity/Het), gene flow (Nm), and the number of accessions assigned to each sub-population.

Population	Inferred Clusters	Mean Fst	Exp. Het	Nm	No. of Accessions
Pop1	0.618	0.5091	0.2385	0.241	238
Pop2	0.382	0.1412	0.3401	1.52	125

**Table 3:** Analysis of molecular variance (AMOVA) revealing genetic diversity in the Afghan landraces.

Method	Source	df	SS	MS	Est. Var.	%
Model-Based (STRUCTURE)	Among Pops	1	366.5567	366.5567	0.698697	0.29%
	Within Pops	361	86625.44	239.9597	239.9597	99.71%
	Total	362	86992		240.6584	100.00%
Distance-Based (Cluster, DAPC)						
	Among Pops	2	1832.573	916.2863	6.001869	2.47%
	Within Pops	360	85159.43	236.554	236.554	97.53%
	Total	362	86992		242.5558	100.00%
Based on Origin						
	Among Pops	6	377.9731	62.99552	0.955026	4.41%
	Within Pops	356	7356.361	20.66393	20.66393	95.58%
	Total	362	7734.334	21.36556	21.61896	100%

df: degrees of freedom, SS: sum of squares, MS: mean square, Est. Var: estimated variance.

**Table 4:** Genetic distance among the populations by different methods.

Method								
Model-Based (STRUCTURE)		Pop1	Pop2					
	Pop1		0.996					
	Pop2	0.004						
Distance-Based (Cluster, DAPC)								
		Pop1	Pop2	Pop3				
	Pop1		0.977	0.991				
	Pop2	0.023		0.982				
	Pop3	0.009	0.018					
Based on Origin								
		Badakhshan	Baghlan	Kabul	Konarha	Kunduz	Samangan	Takhar
	Badakhshan		0.975	0.880	0.773	0.975	0.783	0.997
	Baghlan	0.026		0.907	0.777	0.993	0.813	0.973
	Kabul	0.128	0.098		0.735	0.896	0.747	0.877
	Konarha	0.257	0.252	0.308		0.768	0.671	0.772
	Kunduz	0.025	0.007	0.109	0.263		0.812	0.976
	Samangan	0.245	0.207	0.292	0.399	0.208		0.780
	Takhar	0.003	0.027	0.132	0.259	0.024	0.249	

Nei’s genetic identity is above the diagonal and genetic distance is below the diagonal.

**Table 5:** Mean of different genetic parameters, including the number of different alleles (Na), the number of effective alleles (Ne), Shannon’s index (I), the diversity index (He), the unbiased diversity index (uHe), and the percentage of polymorphic loci (PPL) in each of the two subpopulations.

Method	Pop	Na	Ne	I	He	uHe	PPL
Model-Based (STRUCTURE)	Pop1	1.998	1.594	0.518	0.346	0.348	99.90%
	Pop2	2.000	1.588	0.514	0.343	0.345	100.00%
	Mean	1.999	1.591	0.516	0.345	0.346	99.95%
Distance-Based (Cluster, DAPC *)							
	Pop1	1.987	1.567	0.496	0.331	0.335	99.20%
	Pop2	1.997	1.593	0.517	0.345	0.347	99.80%
	Pop3	1.998	1.578	0.505	0.337	0.340	99.90%
	Mean	1.994	1.579	0.506	0.338	0.341	99.63%
Based on Origin							
	Badakhshan	1.998	1.584	0.509	0.340	0.343	99.90%
	Baghlan	1.984	1.575	0.503	0.336	0.341	98.90%
	Kabul	1.312	1.362	0.304	0.206	0.231	54.90%
	Konarha	0.358	1.000	0.000	0.000	0.000	0.00%
	Kunduz	1.934	1.556	0.486	0.324	0.331	95.60%
	Samangan	0.359	1.000	0.000	0.000	0.000	0.00%
	Takhar	1.998	1.578	0.505	0.337	0.340	99.90%
	Mean	1.420	1.379	0.330	0.220	0.227	64.17%

*—Discriminant Analysis of Principal Components.

## Supplementary Materials

The following are available online at https://www.mdpi.com/2073-4425/12/3/340/s1. Figure S1: STRUCTURE bar plot at *K* = 2 for 363 landraces used in the study. Table S1: List of bread wheat landraces used in the study, including their province of origin and longitude and latitude coordinates. Table S2: Proportion of membership of each predefined population in each of the two clusters of bread wheat landraces using the STRUCTURE program. Table S3: Average temperature and precipitation of provinces in the north-eastern and central agro-ecological zones of Afghanistan.
