# Supplementary figures and images for: Genetic Diversity and Population Structure Analysis of Triticum aestivum L. Landrace Panel from Afghanistan
Source: Genes (Basel). 2021 Feb 25;12(3):340. doi: 10.3390/genes12030340 (PMC7996569; doi:10.3390/genes12030340)

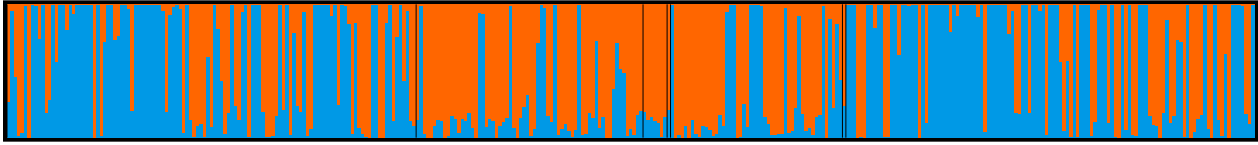

Badakhsha

Baghlan

Kabyl  
Konarha

Kunduz

Samangan

Takhar

Supplement: Supplementary file 1 [file genes-12-00340-s001.zip › Figure S1.pdf]
